# Evaluation of transfer learning in deep convolutional neural network models for cardiac short axis slice classification

**DOI:** 10.1038/s41598-021-81525-9

**Published:** 2021-01-19

**Authors:** Namgyu Ho, Yoon-Chul Kim

**Affiliations:** 1 Department of Computer Science and Engineering, Sogang University, Seoul, Republic of Korea; 2 Clinical Research Institute, Samsung Medical Center, Sungkyunkwan University School of Medicine, 81, Irwon-ro, Gangnam-gu, Seoul, 06351 Republic of Korea

**Keywords:** Biomedical engineering, Magnetic resonance imaging

## Abstract

In computer-aided analysis of cardiac MRI data, segmentations of the left ventricle (LV) and myocardium are performed to quantify LV ejection fraction and LV mass, and they are performed after the identification of a short axis slice coverage, where automatic classification of the slice range of interest is preferable. Standard cardiac image post-processing guidelines indicate the importance of the correct identification of a short axis slice range for accurate quantification. We investigated the feasibility of applying transfer learning of deep convolutional neural networks (CNNs) as a means to automatically classify the short axis slice range, as transfer learning is well suited to medical image data where labeled data is scarce and expensive to obtain. The short axis slice images were classified into out-of-apical, apical-to-basal, and out-of-basal, on the basis of short axis slice location in the LV. We developed a custom user interface to conveniently label image slices into one of the three categories for the generation of training data and evaluated the performance of transfer learning in nine popular deep CNNs. Evaluation with unseen test data indicated that among the CNNs the fine-tuned VGG16 produced the highest values in all evaluation categories considered and appeared to be the most appropriate choice for the cardiac slice range classification.

## Introduction

**Figure 1:**
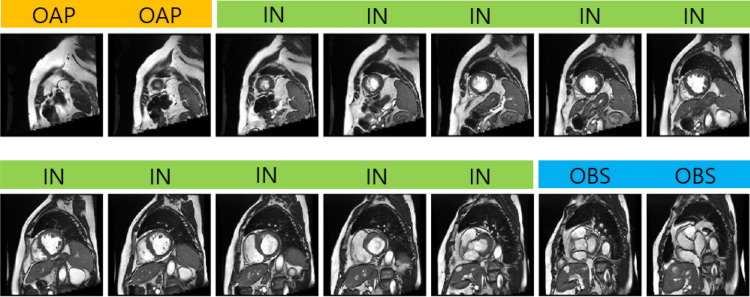
Acquired short axis cine slices from apex to base. Labels are shown on top of each image. The short axis slices corresponding to IN are only valid for quantification such as LV segmentation. This study uses deep learning to automatically classify the short axis slices into one of the three labels: OAP, IN, and OBS. For quantification such as LV ejection fraction, we need to identify short axis slices that correspond to the class IN.

With the recent advances in deep convolutional neural networks (CNNs), there is a growing interest in applying this technology to medical image analysis^1^. Specifically, in the field of cardiac magnetic resonance imaging (MRI), deep CNNs are applied to the left ventricle (LV), right ventricle (RV), and myocardial segmentation for automatic quantification of ejection fraction and myocardial mass^2–4^. The segmentation step is typically preceded by the identification of a short axis slice range, which may require a manual procedure, as a stack of short axis cardiac MR images tends to include slices out of the LV coverage^5^. The standard guidelines provided in Schulz-Menger et al.^6,7^ claim the need for the correct identification of a slice range for diastole and systole for the measurement of LV volume as well as LV mass. Figure 1 shows an example of a stack of short axis slices covering the entire LV with class labeling. Manual identification of a valid short axis slice range is cumbersome and time-consuming, and the automation of the procedure would be desirable for the complete automatization of computer-aided diagnosis in cardiac MRI data.

Deep learning has shown the potential to improve automatic classification of cardiac MR images. Margeta et al. demonstrated the use of fine-tuned CNNs for cardiac MRI scan plane recognition^8^. Their method focused on classifying cardiac MR images into five categories (i.e., 2-chamber, 3-chamber, 4-chamber, LV outflow tract, and short axis). It achieved a high average F1 score, but the study did not investigate the classification of short axis slices into three categories (i.e., out of apical slice, in slice, and out of basal slice) as in our study design. In addition, automatic image quality assessment using a CNN model was demonstrated in Zhang et al.^9^. The method aimed to identify either a missing apical slice or a missing basal slice. Two separate 5-layer CNN models were trained. In cases where the number of images for training is not sufficiently large (e.g., medical image data), training a deep CNN from scratch is likely prone to overfitting. To avoid the problem and to utilize the representational power available from deep CNN models, researchers are increasingly relying on transfer learning^10–12^. Transfer learning has been demonstrated to achieve good classification accuracy as well as efficient model training by reusing trained weights of deep CNNs^13–16^, and has shown remarkable success in image classification from the large-scale ImageNet database.

Early research on transfer learning utilizes pre-trained CNNs as *fixed feature extractors*. Specifically, the pre-trained network takes an image as input and then outputs “off-the-shelf” features from a certain layer in the network. These features are used as input for various classifiers, including random forests, support vector machine (SVM), and dense neural network (DNN). Recent transfer learning methods apply *fine-tuning*, where the convolutional layers of the pre-trained networks are trained further, or *fine-tuned,* with images from a target domain. Fine-tuning has been shown to improve performance on a wide range of classification tasks. Shin et al. used AlexNet^17^ and GoogLeNet^14^ as the base networks and applied transfer learning to the medical image domain^18^. Tajbakhsh et al. demonstrated higher accuracy with fine-tuning in classification performance than a CNN model trained from scratch^19^. Mormont et al. applied transfer learning to digital pathology^20^. They extracted features from the convolutional layers of pre-trained networks and applied various machine learning techniques for classification. They also applied fine-tuning to top-performing base networks to maximize performance. Lee et al. demonstrated high accuracy in bone age assessment with the fine-tuning of GoogLeNet^21^. Kumar et al. proposed an ensemble of fine-tuned AlexNet and GoogLeNet for a variety of medical image modalities^22^. Finally, Gupta et al. applied transfer learning with InceptionV3 as the base deep CNN to coronary plaque detection in computed tomography angiography^23^.

Several major breakthroughs in deep CNN architectures have been achieved for ImageNet classification. In 2014, the VGG architecture demonstrated the potential of deep CNN with convolutional filters of the 3 × 3 size only. GoogLeNet introduced the Inception module, which provided a solution to scale-invariance by concatenating feature maps from convolutional filters of different sizes. GoogLeNet has also set a precedent for future architectures by utilizing repeated structures of novel convolutional blocks. Depth-wise separable convolutions, first explored in Sifre and Mallat^24^, were adopted in many state-of-the-art CNN architectures^15,16,25,26^ in order to enable substantial reduction in computational cost. In 2017, neural architecture search (NAS)^27^ introduced the idea of learning optimal architectures with reinforcement learning, leading to a new family of architectures such as NASNet^26^. Other novel designs such as “dense blocks”^28^ have achieved comparable results in various datasets with a reduced number of parameters.

**Figure 2:**
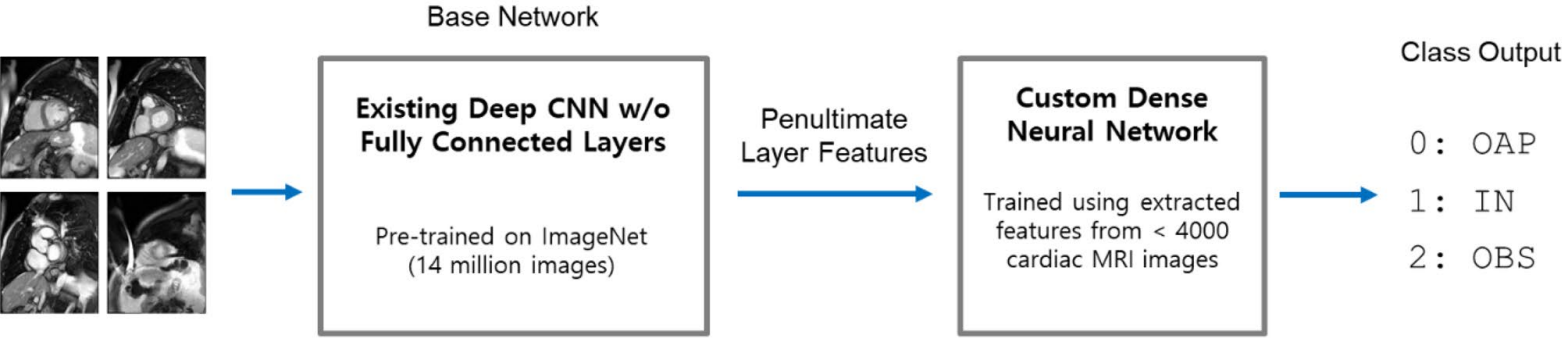
A schematic diagram of transfer learning for the fixed feature extraction setting (i.e., depth = 0). Images are propagated through the convolution layers from the existing deep CNNs (base network) to extract penultimate layer features. The features are used to train a custom DNN classifier. The base network and custom DNN classifier are concatenated to form an end-to-end classification model. Fine-tuned models with depth = 1 are illustrated in the Supplementary Material.

Despite these recent advances in CNN architectures, there is a lack of studies comparing the performance of transfer learning for a wide range of base networks in medical image data. In this study, we evaluated the performance of transfer learning in nine popular CNN architectures in the fixed feature extraction and fine-tuning settings (Fig. 2) for the task of automatic cardiac short axis slice range classification. Fine-tuning was applied to only a subset of convolutional layers as proposed in Tajbakhsh et al.^19^. Classifiers were built to take an image as input and classify it into one of three categories: out-of-apical (OAP), apical-to-basal (IN), and out-of-basal (OBS).

## Results

### Model training and validation

**Figure 3:**
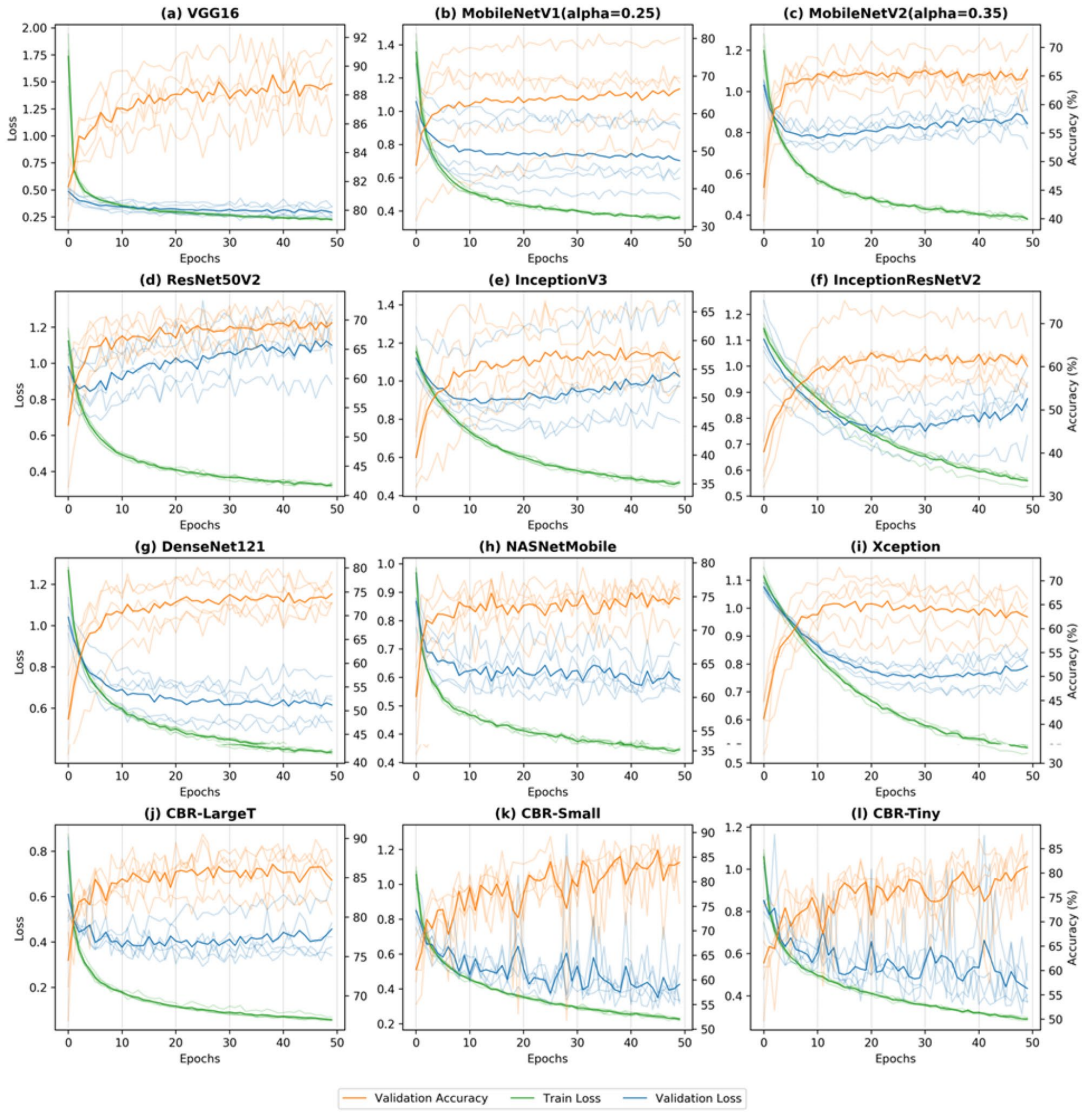
Plots of training and validation loss (green and blue lines, respectively) and validation accuracy (orange lines) in five-fold cross-validation, for a learning rate of 10^–4^. The transparent lines indicate results from individual folds, and the opaque lines show the averages over the five folds.

Figure 3 compares the training history of five-fold cross-validation (CV) for each network in the fixed feature extraction setting. We plotted the history for the fixed learning rate of 10^–4^ to compare the convergence of each model. The VGG16 model showed an exceptionally fast convergence speed, followed by MobileNetV1.

InceptionResNetV2 and Xception showed noticeably slow convergence patterns. The Inception and ResNet models as well as MobileNetV2 showed a high degree of overfitting. We observed a high variance of loss and accuracy metrics across different folds. Among the three baseline models trained from scratch, CBR-LargeT achieved a very low loss on the training set, likely due to a relatively larger number of trainable parameters. The two smaller baseline models (i.e., CBR-Small and CBR-Tiny) did not exhibit patterns of a high degree of overfitting in the validation loss, although the loss curves showed a large degree of fluctuation with respect to epochs.

### Computation time

Table 1 lists the computation time for hyperparameter search in training of the nine models in the fine-tuning setting (depth = 1), where three different learning rates (LRs) and five-fold CVs were performed for each model with 50 epochs. Training and validation were performed on a single GPU (NVIDIA Quadro P5000, 16 GB memory). MobileNetV2 was the fastest in training with a total time of 154 min, and ResNet50V2 was the 2nd place with a total time of 171 min. MobileNetV1, NASNetMobile, DenseNet121, and VGG16 were the next models in ascending order of the computation time. InceptionResNetV2 and Xception were the two worst performed models, taking 746 and 380 min, respectively. Notably, InceptionResNetV2 produced a GPU memory error with the default batch size of 32, and we reduced the batch size down to 8. This might have adversely affected the computation performance.

**Table 1:** Comparison of computation time measured during model training and validation.

Base network	Time (min)^a^
MobileNetV1 (a = 0.25)	172
MobileNetV2 (a = 0.35)	154
VGG16	217
InceptionV3	308
ResNet50V2	171
InceptionResNetV2	746
DenseNet121	212
NASNet Mobile	181
Xception	380

^a^Time indicates computation time taken to complete the three learning rates, where for each learning rate the five-fold cross-validation was performed.

### Evaluation on test data

Table 2 displays the evaluation results of the models for the nine deep CNN models and three baseline CBR models. The fine-tuned VGG16 model showed the highest accuracy of 0.90 and soft accuracy of 0.97, outperforming the best baseline model, CBR-Small, which achieved an accuracy of 0.86 and a soft accuracy of 0.95. The majority of models were similar in accuracy scores ranging from 0.81 to 0.90, with the exception of MobileNetV2 and NasNet Mobile, which ranged from 0.65 to 0.73. F1 scores were the highest for the fine-tuned VGG16. Micro-averaged area-under-the-curve (AUC) scores showed patterns similar to accuracy. The fine-tuned VGG16 and InceptionResNetV2 obtained the top micro-averaged AUC scores of 0.98, while MobileNetV2 and NasNet Mobile showed the lowest scores. For F1 scores, we observed a high level of variance in the outer slices (i.e., OAP and OBS), with OAP ranging 0.36–0.81 and OBS ranging 0.46–0.80. For models with low F1 scores in the outer slices, InceptionV3 scored F1 values of 0.36–0.38 for OAP, and MobileNetV2 scored F1 values of 0.46–0.52 for OBS.

**Table 2:** Test results of the nine models with different depth levels as well as three baseline models.

Base network	Depth^a^	F1 score			AUC^b^	Accuracy	Soft accuracy
		OAP	IN	OBS			
MobileNetV1 (a = 0.25)	0	0.56	0.90	0.73	0.97	0.85	0.95
MobileNetV1 (a = 0.25)	1	0.64	0.90	0.74	0.97	0.85	0.95
MobileNetV2 (a = 0.35)	0	0.56	0.78	0.46	0.86	0.67	0.76
MobileNetV2 (a = 0.35)	1	0.77	0.81	0.52	0.89	0.73	0.82
VGG16	0	0.78	0.91	0.76	0.97	0.86	0.94
VGG16	1	**0.81**	**0.93**	**0.80**	**0.98**	**0.90**	**0.97**
InceptionV3	0	0.38	0.88	0.68	0.95	0.81	0.92
InceptionV3	1	0.36	0.87	0.67	0.95	0.81	0.92
ResNet50V2	0	0.62	0.90	0.70	0.96	0.84	0.94
ResNet50V2	1	0.71	0.90	0.72	0.97	0.85	0.94
InceptionResNetV2	0	0.66	0.88	0.65	0.96	0.83	0.93
InceptionResNetV2	1	0.78	0.92	0.79	**0.98**	0.89	0.96
DenseNet121	0	0.67	0.90	0.73	0.97	0.85	0.93
DenseNet121	1	0.71	0.90	0.74	0.97	0.86	0.93
NASNet Mobile	0	0.57	0.78	0.58	0.88	0.70	0.80
NASNet Mobile	1	0.49	0.71	0.63	0.84	0.65	0.76
Xception	0	0.51	0.88	0.65	0.95	0.82	0.93
Xception	1	0.63	0.89	0.69	0.96	0.84	0.93
CBR-LargeT	–	0.69	0.90	0.77	0.96	0.85	0.93
CBR-Small	–	0.75	0.91	0.72	0.97	0.86	0.95
CBR-Tiny	–	0.61	0.84	0.74	0.92	0.78	0.88

Bold text indicates the highest value among the models.

*CBR* convolution, batch-normalization, ReLu-activation^29^.

^a^The definition of depth is provided in the Supplementary Material.

^b^Micro-averaged AUC score.

**Figure 4:**
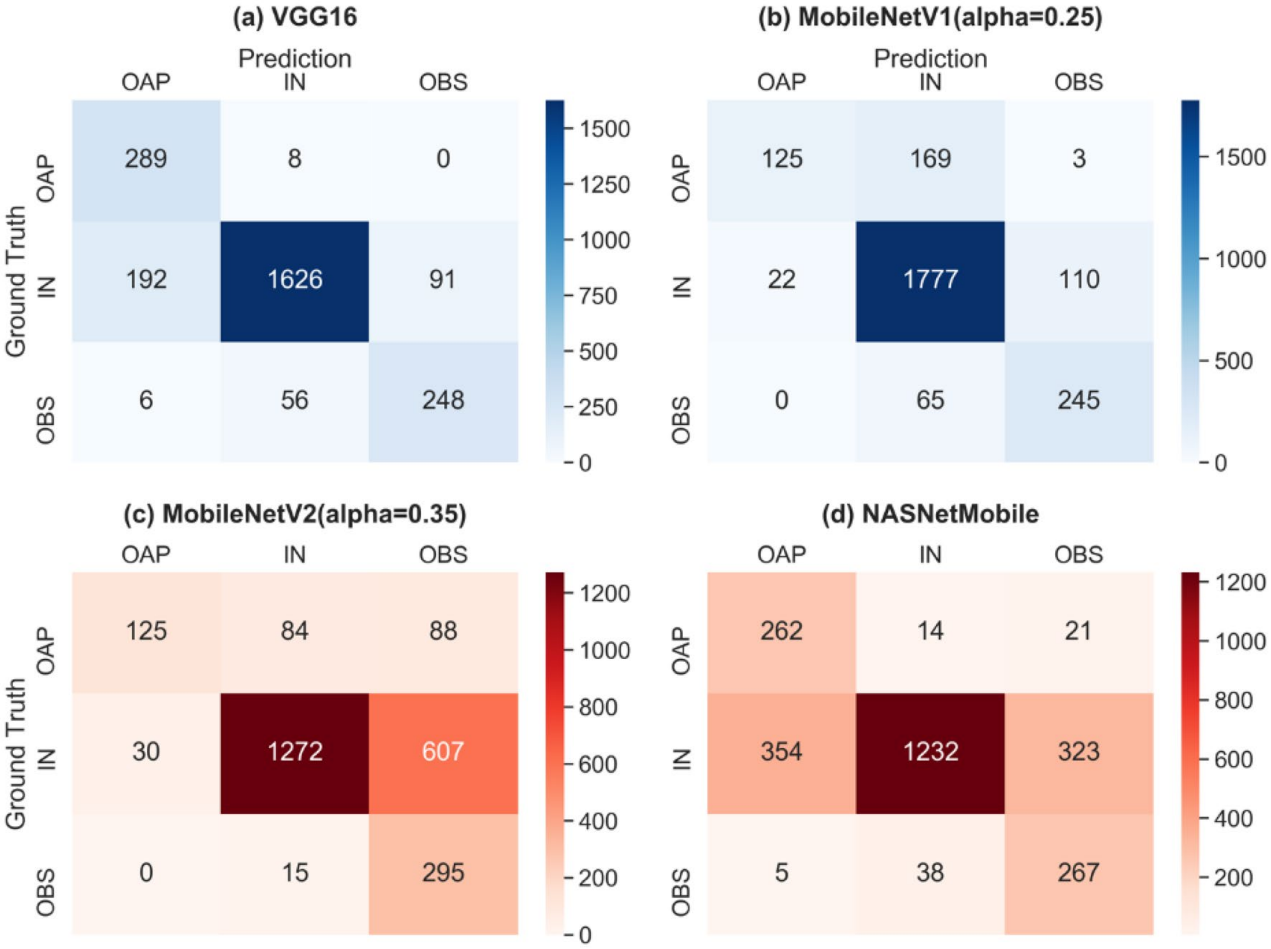
Confusion matrices of test prediction results from the good (**a**,**b**) and poor (**c**,**d**) performing models.

**Figure 5:**
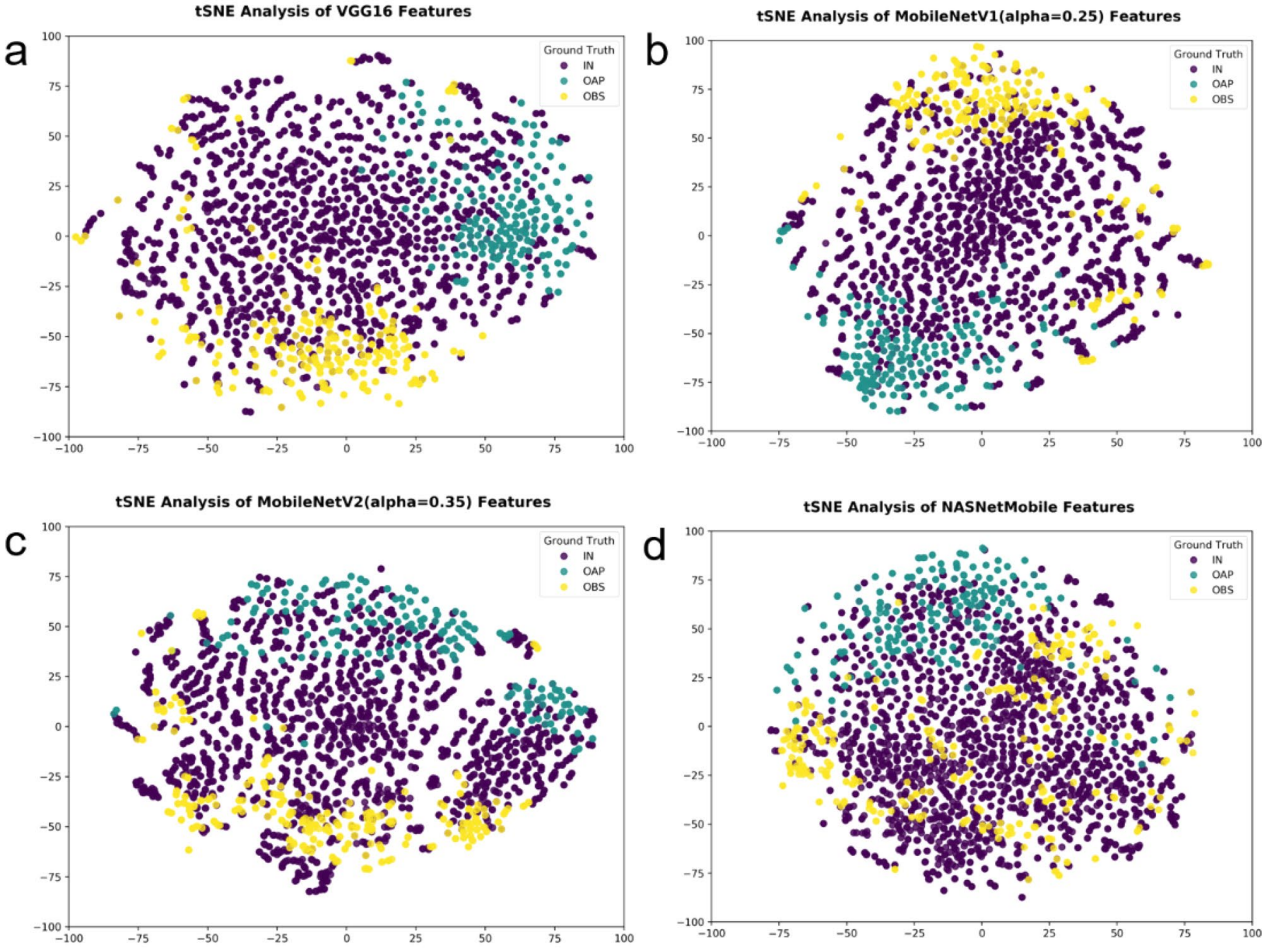
tSNE visualization by dimensionality reduction of the penultimate features. The test data are shown in colors for different classes. The intra-class samples in the good-performance models (**a**,**b**) tend to be more clustered than those in the poor-performance models (**c**,**d**).

Figure 4 shows the visualization of confusion matrices using the scikit-learn’s confusion_matrix function^30^. The class prediction results are shown for two good performing models VGG16 and MobileNetV1 and two poor performing models MobileNetV2 and NASNet Mobile. VGG16 often misclassified IN slices as OAP, while MobileNetV1 misidentified OAP slices as IN. For the poor performing cases, MobileNetV2 often misclassified IN slices as OBS, and NASNet Mobile misclassified IN slices as either OAP or OBS. In Fig. 5, the t-Distributed Stochastic Neighbor Embedding (tSNE) analysis for dimensionality reduction indicates that in the good-performance models (a-b), the intra-class samples tend to be more clustered than those in the poor-performance models (c-d).

**Figure 6:**
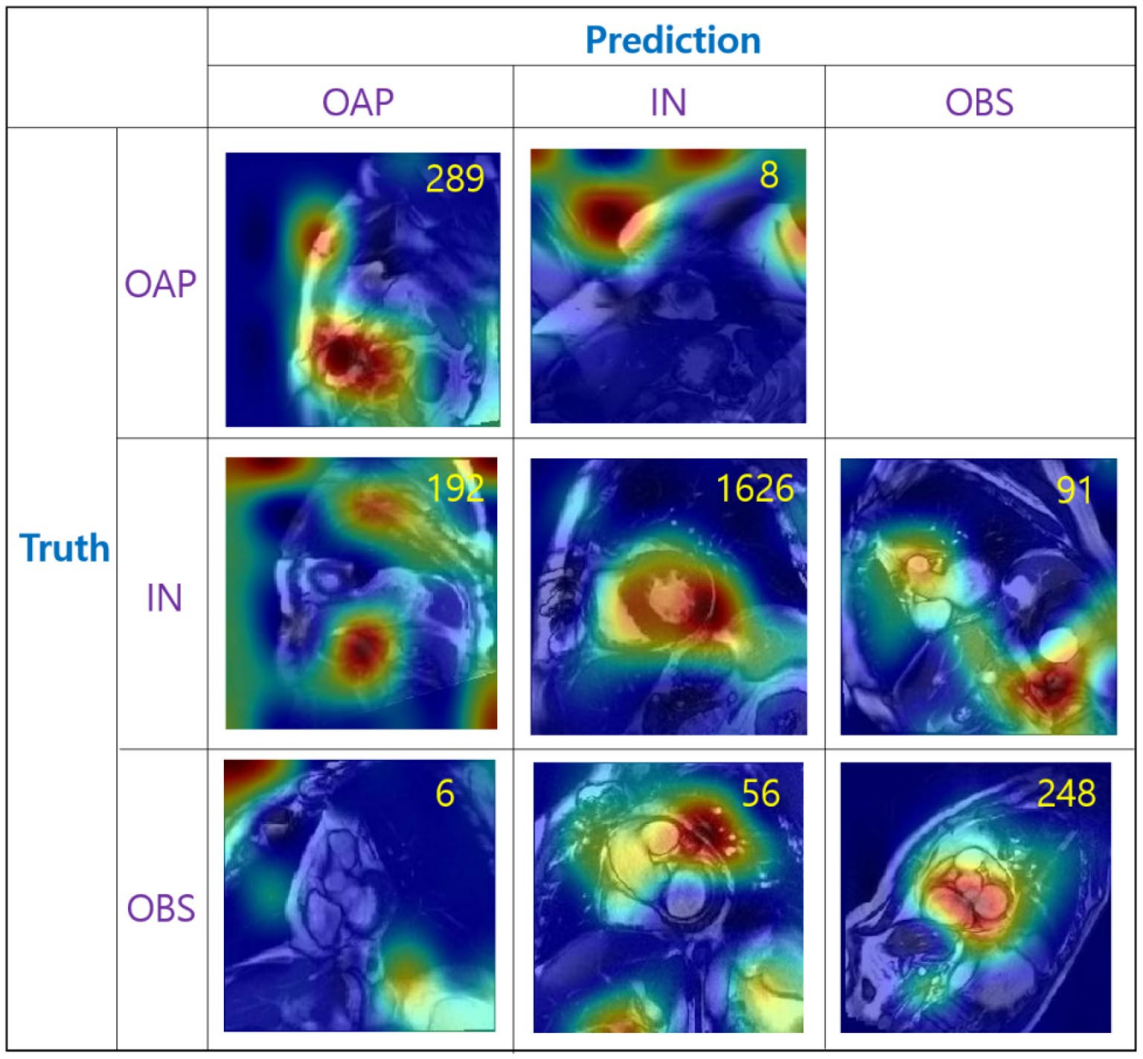
Representative examples of heat map patterns generated from the VGG16 model with test images as input. The yellow number shown in each cell indicates the number of images corresponding to the cell. There were no cases corresponding to the cell (Truth, Prediction) = (OAP, OBS).

In Fig. 6, the class activation maps generated from VGG16 are shown for the different combinations of the (Truth class, Prediction class) cases. We used the gradient based Grad-CAM visualization method by Selvaraju et al.^31^. It is noted that the model made correct predictions when the myocardium was accurately localized. For the incorrect cases, the model appears focusing on regions outside the myocardium. The incorrect cases such as (Truth, Prediction) = (IN, OAP), (IN, OBS), (OBS, IN) show the activation peaks in the regions that appear visually similar to the myocardium, indicating that the model attempts to identify the myocardium. Some incorrect cases such as (Truth, Prediction) = (OBS, OAP), (OAP, IN) show strong activation patterns in regions near the borders of the images.

## Discussion

In this study, we applied transfer learning to the task of automatic slice range classification in cardiac short axis images. The F1 score, micro-averaged AUC, accuracy, and soft accuracy of deep CNN models were compared, and the fine-tuned VGG16 resulted in the highest values in all six evaluation categories (Table 2). Our study suggests that even the “light” MobileNetV1 and CBR models, which have relatively small numbers of parameters, show moderate accuracy compared to VGG16 and appear as a suboptimal yet feasible choice.

Numerous studies explored the effects of transfer learning^18,19,23,29,32,33^, but a few of them provided comparisons of as many recent deep CNNs as in our study. Notably, Kornblith et al.^32^ conducted a more expanded comparison of 16 different CNNs and found a strong correlation between ImageNet accuracy and transfer accuracy, but this was not observed in our study. It is noted that the datasets used in Kornblith et al.^32^ are natural images (e.g., food, cars, flowers, etc.) rather than medical images. Burgos-Artizzu et al. evaluated 19 deep CNN models including VGG16 for the classification of fetal ultrasound images and reported that DenseNet-169 was the best performing model in terms of top-1 error^34^. This suggests that the transferability of ImageNet models is application-dependent and can vary depending on the difference between source domain and target domain.

We explored the fixed feature extraction setting (i.e., depth = 0) and the fine-tuning of the last convolutional layer (i.e., depth = 1) for transfer learning. The fine-tuning setting produced slightly higher classification accuracy in most of the base deep neural networks than the fixed feature extraction setting. To further improve performance, we may consider exploring the fine-tuning of more layers down to the first layer, but this was not pursued in the current study as VGG16 outscored the other networks in terms of classification accuracy. We noted that the layer-wise fine-tuning scheme had to be customized with care for most of the deep CNNs, since they involve repetitions of complex convolutional blocks rather than a simple sequential stack of layers as in VGG16.

Our approach is based on the individual classification of a single short axis slice image, but it is worth considering methods that take advantage of the information of adjacent slices, because a stack of contiguous short axis slices from apex to base is acquired in practice. There are several approaches to exploiting the information of adjacent slices:

- Recurrent neural networks (RNN): Often used for time-series modeling, RNNs can effectively encode information from previous and/or subsequent slices, given an ordered sequence of image slices from a single patient. Its use has been demonstrated in cardiac segmentation^35^ and intracranial hemorrhage subtype classification^36,37^.
- Three-dimensional (3D) CNN: Using a set of slices as volumetric data, it is possible to extract features directly from the volume using 3D convolutions as explored in Isensee et al.^38^.
- 2D/3D CNN: It is also possible to concatenate multiple slices into a single two-dimensional (2D) image with multiple channels and build a 2D/3D CNN to utilize information from adjacent slices through channel-wise correlations as demonstrated in Shan et al.^39^.

One may consider applying transfer learning to the methods above. For RNN-based networks, pre-trained ImageNet models can be used for feature extraction, and the recurrent layers can be appended on top, similar to the DNN layers in our fine-tuned models. For the 2D/3D CNN approach, the weights from pre-trained 2D CNNs can be transferred to train a 3D CNN model^39^. These methods are worthy of investigation in future studies.

A limitation of our study is that rigorous comparisons were not performed with other related studies. To our knowledge, there have been no studies classifying cardiac short axis slices into the three categories. However, it is noted that a similar study was performed by Zhang et al.^9^. They focused on developing a deep CNN model to identify whether the basal slice (or the apical slice) is missing or not. Unlike our approach, they attempted to develop two separate models, one for the identification of the basal slice and the other for the identification of the apical slice. In addition, data sources for model training and validation are different. The UK Biobank data^40^ were used in Zhang et al.’s work, while the Cardiac Atlas Project data^41^ were used in our study.

In summary, we have investigated the feasibility of transfer learning for automatic categorization of cardiac cine short axis slices by evaluating nine popular deep CNNs. The evaluation of unseen test data indicated that the fine-tuned VGG16 provided the highest values in all evaluation categories considered and appeared to be the most appropriate choice for the classification of a cardiac cine MRI short axis slice range.

## Methods

### Code

Code related to this study is available at https://github.com/itsnamgyu/cardiac-research.

### Dataset

In the present study, we used publicly available data from the left ventricular (LV) cardiac MRI segmentation challenge^41^. The data consisted of cardiac cine image series of short axis and long axis orientations from 200 subjects, where there were 20–30 dynamic frames per slice and 10–16 slices per subject. Out of 20–30 dynamic frames, we only considered two frames: one end-systolic frame and one end-diastolic frame. The stacks of short axis slices from one group of 100 subjects were considered for training/validation, and the stacks of short axis slices from the other group of 100 subjects were considered for testing. The total numbers of images per class are shown in Supplementary Table S1.

### Data labeling

To label the images, we developed a custom user interface, implemented using the Matplotlib^42^ library for Python, for image slice labeling in a diastolic and a systolic frame for all subjects. The interface loads all the cardiac short axis image location information along with corresponding patient identification numbers. For labeling, the user interacts with the layout to classify each short axis slice into one of the following three categories: (1) out-of-apical (OAP), (2) apical-to-basal (IN), and (3) out-of-basal (OBS). OAP was defined as the slice that shows no appearance of the LV blood pool. IN was defined as the slice that shows clear appearances of the myocardium and LV blood pool. OBS was defined as the slice above the most basal slice, which is characterized by a small crescent of basal lateral myocardium and no discernable LV blood pool^6^. The labeling results are saved upon closing the interface. They are saved in an internal metadata file, which is reloaded when the user resumes the manual labeling task.

### Image preprocessing and augmentation

As shown in Supplementary Table S1, the sample size of the IN class is significantly larger than the OAP and OBS classes. To overcome the class imbalance issue, we oversampled the slices corresponding to the OAP and OBS classes by a factor of 6. We used a simple augmentation scheme, which applied random rotations between − 45 and 45 degrees for each image (Supplementary Figure S1). The classification task involves the examination of the myocardium, which is positioned around the center of the images. To reduce unwanted features in the image data, we cropped the outer 40% of both the vertical and horizontal axes of each image. The image cropping retained the myocardial region of interest in all of the images. To prevent data leakage, the augmentation was applied after the data split for the cross-validation. For the evaluation, we applied the same procedure of cropping to the input images.

### Model training and validation

We considered nine well-established CNN architectures for transfer learning. Supplementary Table S2 lists the networks considered in our study, including their capacities, the number of penultimate features, and the ImageNet accuracy scores. We applied transfer learning to cardiac MR images in the fixed feature extraction and fine-tuning settings. For the fixed feature extraction setting, we used the penultimate features from the convolutional base of the nine CNN models as an input to a custom deep neural network (DNN) classifier. For the fine-tuning setting, we considered only a subset of convolutional layers, following the suggestion of a layer-wise fine-tuning scheme proposed in Tajbakhsh et al.^19^. We also trained three baseline models from scratch for comparison. We adopted models from the CBR (convolution, batch-normalization, ReLu activation) family of CNN architectures introduced in Raghu et al.^29^, which follows conventional design of CNN architectures. We considered the CBR-LargeT, CBR-Small, and CBR-Tiny models, which are small in network size (approximately 1/3 to 1/60 of the size of standard deep CNN architectures used for ImageNet classification).

All pre-trained CNN models take natural images, with three color channels, as input, but our study deals with grayscale MRI images. For compatibility, we simply duplicated the grayscale channel to synthesize RGB images. This has the same effect as averaging out the color channels of the convolutional kernels in the first convolutional layer of each network.

In the fixed feature extraction setting, we appended our custom DNN classifier to the existing base networks and froze the base convolutional layers during training. We removed the existing fully connected classifier layers and replaced them with a DNN classifier. The DNN classifier consisted of a dense layer with 256 nodes and ReLU activation, a dropout layer with a dropout rate of 0.5, and a dense layer with 3 nodes and softmax activation. The final layer has three output nodes that correspond to the three classes in our classification task: OAP, IN, OBS. We used pre-trained weights provided by the Keras Applications library^43^.

We used a similar approach for the fine-tuning setting, where we unfroze only those layers considered for further training. We applied fine-tuning to all nine base architectures, using the final models obtained from the fixed feature extraction stage. We selected the layers for fine-tuning based on the individual designs of the base architectures. All architectures are comprised of a series of unique convolutional blocks. We considered the last convolutional block of each network for fine-tuning. The diagrams of the last convolutional blocks for the nine neural networks are shown in the Supplementary Material.

Model development was performed on a single GPU (NVIDIA Quadro P5000, 16 GB memory). To train the network, we used mini-batch gradient descent optimization with a batch size of 32, a decay of 10^–6^, and Nesterov momentum of 0.9. For hyperparameter optimization, we considered three learning rates. Learning rates were 10^–2^, 10^–3^, and 10^–4^ for the fixed feature extraction setting, while they were 10^–3^, 10^–4^, and 10^–5^ for the fine-tuning setting. For our baseline CBR models, we considered learning rates of 10^–2^, 10^–3^, and 10^–4^.

For a given learning rate, we performed a five-fold cross-validation and divided the training/validation set into five distinct subsets, each containing image slices from 20 patients. For each fold, one subset was used for validation and the remaining four were used for training. Hence, a total of five models were trained and validated to evaluate the performance of a single parameter choice. We trained each model for 50 epochs and selected an appropriate epoch number based on manual inspection of the average validation accuracy curve. After setting the epoch number and learning rate, we trained a final model on the entire training/validation set. We used this process in both the fixed feature extraction and fine-tuning settings for the deep CNN models, as well as in the training of the baseline CBR models.

### Evaluation

A total of 21 final models were evaluated: 9 models obtained by training a custom DNN classifier on top of base CNNs used for the fixed feature extraction setting, and 9 models obtained through the fine-tuning, and 3 baseline models trained from scratch. Performance was evaluated against a test dataset of 100 patients that was held out during the model development. The total number of test images per class is shown in Supplementary Table S3. To evaluate multi-class classification performance, we used the following metrics: F1 score, accuracy, micro-averaged AUC score, and soft accuracy. We defined soft accuracy as an alternative measure of accuracy, where each prediction is considered correct if it matches the class of the current slice or either of the adjacent slices. We introduced this metric to account for the inherent interobserver variability in slice-range classification. Given a continuous set of short axis MRI slices, the task of determining N slice ranges is equivalent to determining N-1 boundaries. During the slice classification, we noticed that misidentifying these boundaries by one slice often yields acceptable results. Manual inspection of incorrect model predictions revealed that most errors fell into the boundary cases.

To evaluate the performance on individual classes, we considered the one-vs-all classification task for each class. For example, by regrouping the classes into OAP and non-OAP (IN, OBS), we obtain a binary classification for the OAP class. We applied this to all three classes and evaluated the precision, recall, AUC for each binary classification. The F1 score was defined as 2 × (precision × recall)/(precision + recall). The micro-averaged AUC score was calculated using the weighted average of these AUC scores, based on the number of images in each class.

For the visualization of clustering of the intra-class samples, we applied the t-Distributed Stochastic Neighbor Embedding (t-SNE) method^44^ to the penultimate features (Supplementary Table S2) of the CNN models and reduced the dimensions to 2. Each data point was colored depending on the class type (i.e., OAP, IN, OBS) for the visualization in a 2D space.

As shown in Supplementary Figure S2, we implemented two modes in our graphical user interface that (1) displays probability scores for a given set of short axis slices per subject and (2) shows class activation maps for the predicted class of each slice. The class activation maps (CAMs) were generated using the gradient based Grad-CAM technique^31^. For our models, the CAMs correspond to the weighted sum of each feature map outputted from the base network, based on their contribution to the final output prediction.

## Supplementary Information

Supplementary Information
