# Supplementary material for: Evaluation of transfer learning in deep convolutional neural network models for cardiac short axis slice classification
Source: Sci Rep. 2021 Jan 19;11:1839. doi: 10.1038/s41598-021-81525-9 (PMC7815707; doi:10.1038/s41598-021-81525-9)
Supplement: Supplementary file 1 — Supplementary Information [file 41598_2021_81525_MOESM1_ESM.docx]

**Supplementary Material**

**Evaluation of transfer learning in deep convolutional neural network models for cardiac short axis slice classification**

Namgyu Ho^1^ and Yoon-Chul Kim^2*^

^1^Department of Computer Science and Engineering, Sogang University, Seoul, Republic of Korea, ^2^Clinical Research Institute, Samsung Medical Center, Sungkyunkwan University School of Medicine, Seoul, Republic of Korea

^*^Correspondence to:

Yoon-Chul Kim, PhD

Samsung Medical Center, Sungkyunkwan University School of Medicine

81, Irwon-ro, Gangnam-gu, 06351, Seoul, Republic of Korea

Tel: +82-2-2148-7140

Email: yoonckim1@gmail.com

Running Title: Transfer Learning for Cardiac Slice Classification

**Supplementary Material**

**Supplementary Table 1.** List of the number of training and validation images, by class. The left side refers to the number of images from the original dataset, and the right side refers to the number of images after data augmentation.

|  | Original | | | | Oversampled | | | |
| --- | --- | --- | --- | --- | --- | --- | --- | --- |
|  | OAP | IN | OBS | TOTAL | OAP | IN | OBS | TOTAL |
| Image Count | 386 | 1808 | 328 | 2522 | 1930 | 1808 | 1640 | 5378 |
| Percentages | 15.3 | 71.7 | 13.0 | 100 | 35.9 | 33.6 | 30.5 | 100 |
| Images Per Patient | 3.9 | 18.1 | 3.3 | 25.2 | 19.3 | 18.1 | 16.4 | 53.8 |

**Supplementary Table 2.** Deep CNN specifications.

| Base Network | Input Size | Params | Depth | Penultimate Features | ImageNet Accuracy | |
| --- | --- | --- | --- | --- | --- | --- |
|  |  |  |  |  | Top1 | Top5 |
| MobileNetV1(a=0.25)[^1^](#_ENREF_1) | 224x224 | 0.5M | 28 | 256 | 0.506 | - |
| MobileNetV2(a=0.35)[^2^](#_ENREF_2) | 224x224 | 1.7M | 63 | 448 | 0.603 | 0.829 |
| VGG16[^3^](#_ENREF_3) | 224x224 | 138.3M | 16 | 512 | 0.713 | 0.901 |
| InceptionV3[^4^](#_ENREF_4) | 299x299 | 23.9M | 47** | 2048 | 0.779 | 0.937 |
| ResNet50V2[^5^](#_ENREF_5) | 224x224 | 25.6M | 50 | 2048 | 0.760 | 0.930 |
| InceptionResNetV2[^6^](#_ENREF_6) | 299x299 | 55.9M | 96** | 1792 | 0.803 | 0.953 |
| DenseNet121[^7^](#_ENREF_7) | 224x224 | 8.1M | 121 | 1024 | 0.750 | 0.923 |
| NASNet Mobile[^8^](#_ENREF_8)* | 224x224 | 5.3M | 20* | 1056 | 0.744 | 0.919 |
| Xception^[9](#_ENREF_9)^ | 299x299 | 22.9M | 37*** | 2048 | 0.790 | 0.945 |

The depth values reported in the original papers of these architectures include the number of weight layers–i.e., convolutional layers and fully connected layers.

*NASNet-A, 4 @1056 variant, as denoted in the original paper.

**The original papers for these architectures do not provide the information of the depths of the neural networks. This may be due to the inclusion of parallel paths of convolutional layers in Inception modules and NASNet’s convolutional cells. We have determined the depths of these networks by selecting the maximum depth of these parallel paths.

***We have added 1 to the depth value reported in the original paper, as it does not account for the final fully connected layer.

**Supplementary Table 3.** List of the number of test images for each class.

|  | OAP | IN | OBS | TOTAL |
| --- | --- | --- | --- | --- |
| Image Count | 297 | 1909 | 310 | 2516 |
| Percentages | 11.8 | 75.9 | 12.3 | 100 |
| Images Per Patient | 3.0 | 19.1 | 3.1 | 25.2 |


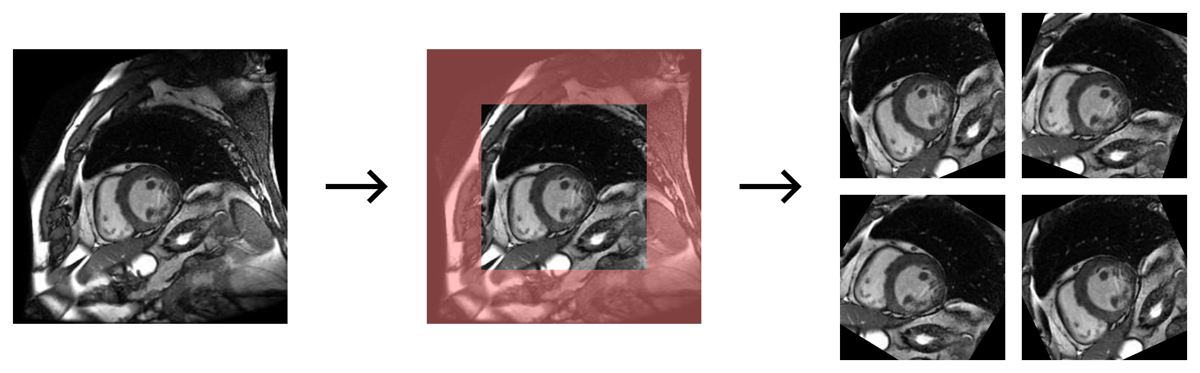


**Supplementary Figure 1.** Image pre-processing steps involving center cropping and random rotations. Data augmentation is performed by random rotations of the image.


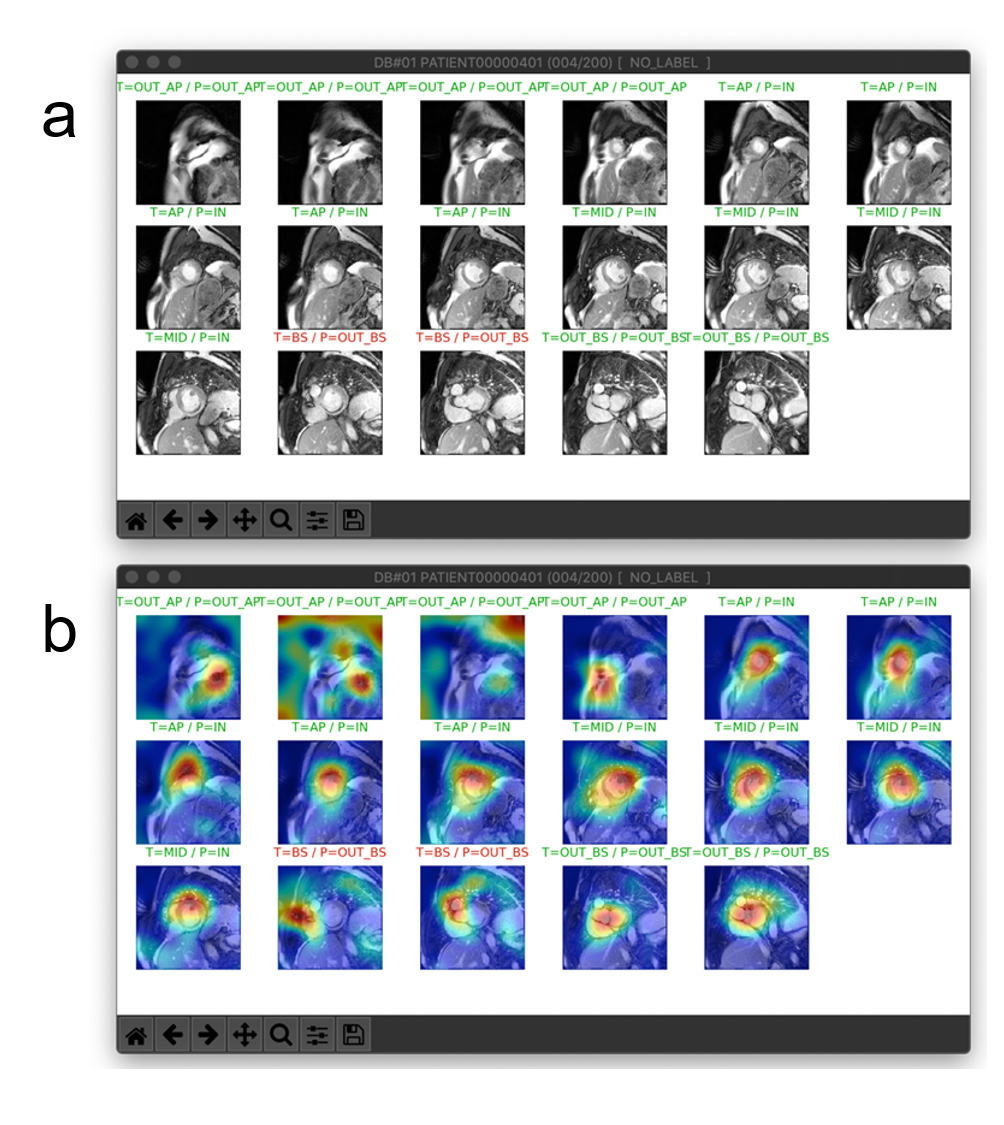
**Supplementary Figure 2.** Modules for data labeling and individual predictions. (a) A data labeler for supervised learning. Option is provided to show output predictions from developed models. Probability scores are shown on each image. The green text indicates correct classification while the red text indicates incorrect classification. (b) Class activation maps for predicted classes obtained using Grad-CAM method [^10^](#_ENREF_10).

**Fine-Tuning Architectures**

1. **MobileNetV1(a=0.25)**

The MobileNetV1 architecture is comprised of a single 3 × 3 standard convolution for low-level feature extraction followed by 13 depthwise-separable (separable) convolutions. Global average pooling is performed on the penultimate feature maps, and it is common for all architectures considered in our study, with the exception of VGG16. For fine-tuning, we tuned the final separable convolution. Note that each separable convolution is comprised of one 3 × 3 depthwise convolution layer and one 1 × 1 pointwise convolution layer.

**Figure A1.** A simple illustration of the MobilenetV1 architecture, before global average pooling. The lines represent the weight freezing boundaries in the fixed feature extraction setting referred to as depth = 0 (red line) and the fine-tuning setting referred to as depth = 1 (green line), respectively.


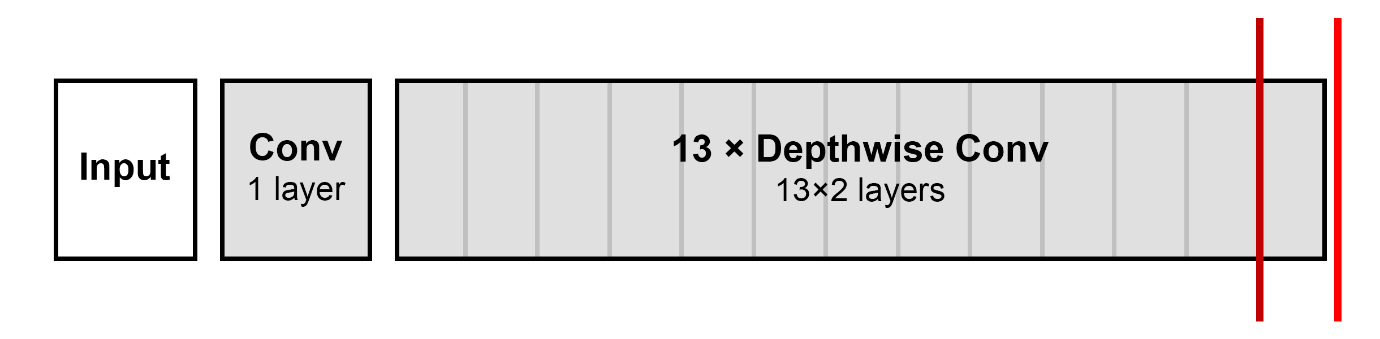


1. **MobileNetV2(a=0.35)**

The MobileNetV2 architecture is comprised of a single 3 × 3 convolution for low-level feature extraction, a series of 17 bottleneck residual blocks, and a final 1 × 1 pointwise convolution which expands the number of final feature maps. We considered the final bottleneck residual block for fine-tuning.

**Figure A2.** A simple illustration of the MobileNetV2 architecture, before global average pooling. The lines indicate weight freezing boundaries, where depth = 1 and depth = 0 are indicated by green and red colors, respectively.


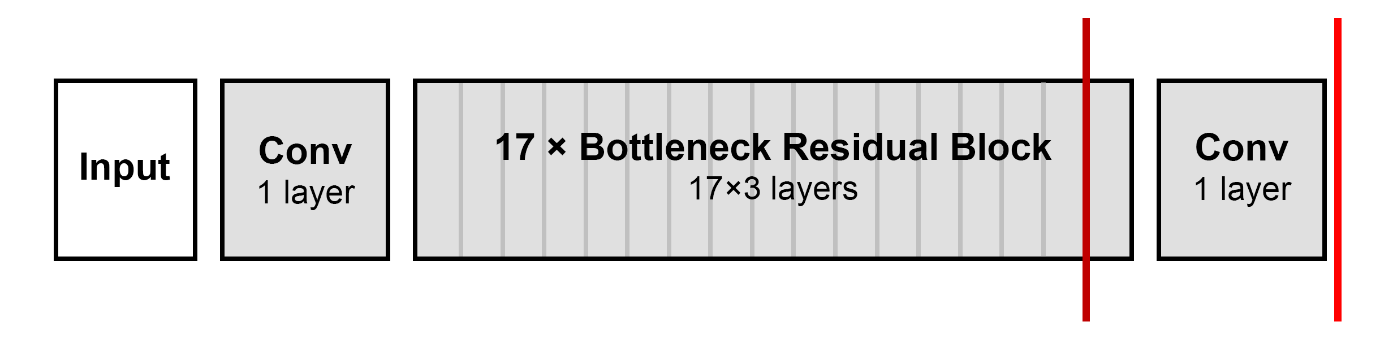


1. **VGG16**

The VGG16 architecture is comprised of 5 convolutional blocks, each containing 2, 2, 3, 3, 3 convolutional layers respectively. 2 × 2 max pooling is performed after each convolutional block. Unlike all other networks considered in our study, global average pooling is not applied to the penultimate features, and the final classifier consists of three dense layers instead of one. In our study, we commonly use global average pooling and the same two-layer DNN classifier for all networks including VGG16 for fair comparison.

**Figure A3.** A simple illustration of the VGG16 architecture, before dense layers. The lines indicate weight freezing boundaries, where depth = 1 and depth = 0 are indicated by green and red colors, respectively.


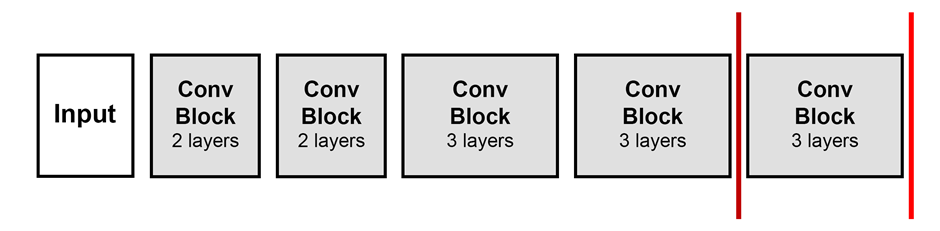


1. **InceptionV3**

The InceptionV3 architecture is comprised of 6 convolutional layers at the base for low-level feature extraction followed by a series of convolutional blocks called “Inception” modules. Three distinct variants of Inception modules are each repeated 3, 5, 2 times respectively. For fine-tuning, we only considered the last instance of the final Inception module variant.

**Figure A4.** A simple illustration of the InceptionV3 architecture, before global average pooling. The lines indicate weight freezing boundaries, where depth = 1 and depth = 0 are indicated by green and red colors, respectively.


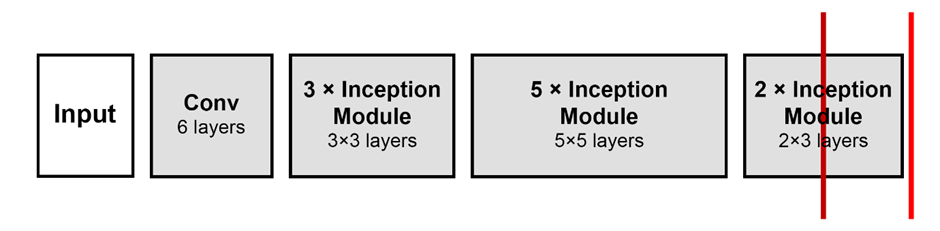


1. **ResNet50V2**

The ResNet architecture uses a single convolutional layer for early feature extraction, followed by a repetition of four different “building blocks”. In the 50-layer variant, each building block is comprised of three convolutional layers, each with residual connections between the input and output. Each block type is repeated 3, 4, 4, 3 times respectively. ResNetV2 introduces improvements to the aforementioned residual connections while maintaining the overall structure of the original architecture variants. We considered only the final building block instance for fine-tuning on ResNet50V2.

**Figure A5.** A simple illustration of the ResNet50V2 architecture, before global average pooling. The lines indicate weight freezing boundaries, where depth = 1 and depth = 0 are indicated by green and red colors, respectively.


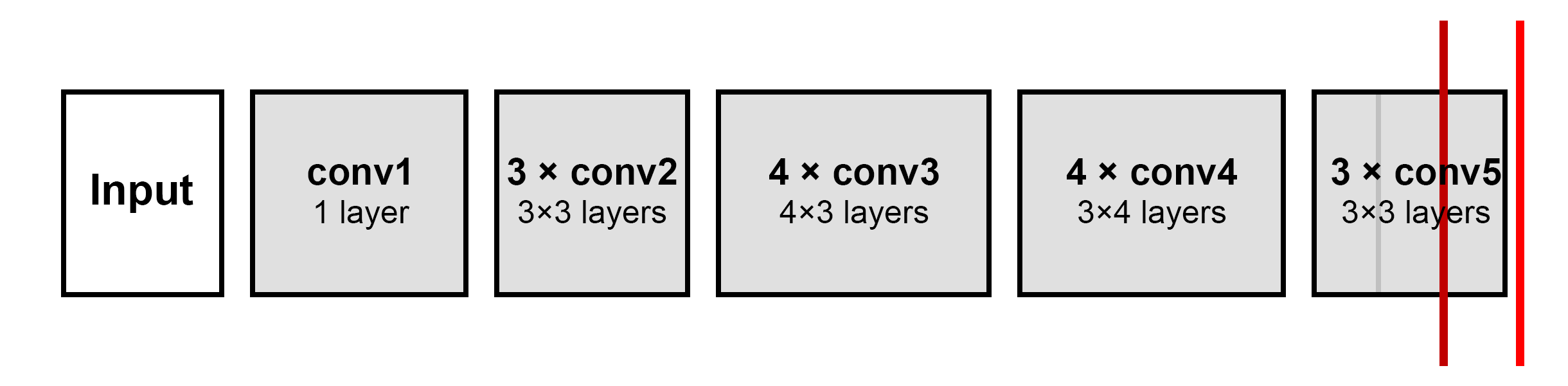


1. **InceptionResNetV2**

InceptionResNetV2, which was introduced alongside InceptionV4 and InceptionResNetV1, improves on InceptionV3 by incorporating residual connections to Inception modules. InceptionResNetV2 uses 8 convolutional layers for early feature extraction, and a repetition of three distinct Inception module designs, each repeated 5, 10, 5 times respectively. The new architectures also introduce “reduction blocks” placed between each variant of Inception modules for feature map reduction. Similar to InceptionV3, we only considered the last instance of the final Inception module variant (denoted “Inception-Resnet-C” in the original paper) for fine-tuning.

**Figure A6.** A simple illustration of the InceptionResNetV2 architecture, before global average pooling. The unlabeled blocks represent reduction blocks (“Reduction-A” and “Reduction-B” as denoted in the original paper). The lines indicate weight freezing boundaries, where depth = 1 and depth = 0 are indicated by green and red colors, respectively.


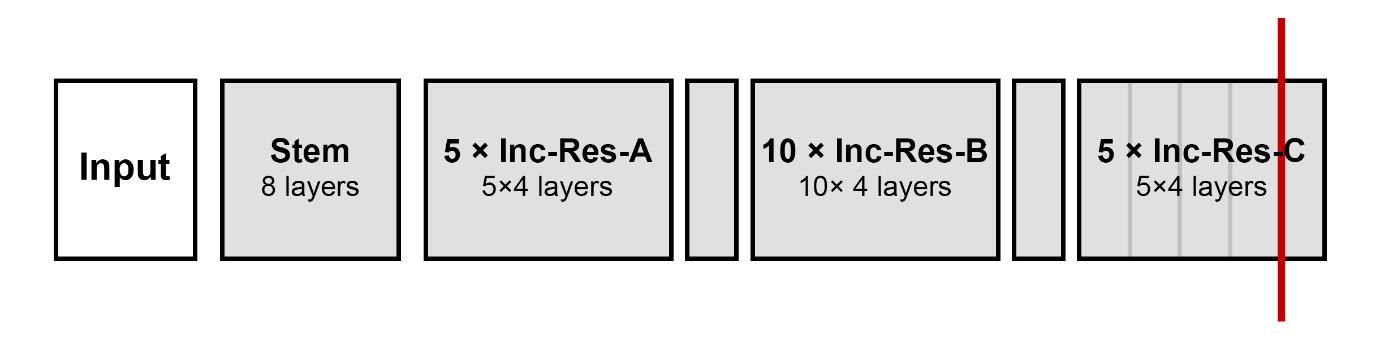


1. **DenseNet121**

The DenseNet architecture is comprised of a single 7 × 7 convolution for early feature extraction, and four “dense blocks” connected by “transition layers”. Each dense block contains a certain number of convolutional layers that are densely connected, i.e., each layer receives the feature maps of all previous layers (within the block) as input. The transition layer is a simple combination of one 1 × 1 convolution followed by 2 × 2 average pooling. We considered the last dense block, which contains 32 convolutional layers, for fine-tuning.

**Figure A7.** A simple illustration of the DenseNet architecture, before global average pooling. The unlabeled blocks represent transition blocks. The lines indicate weight freezing boundaries, where depth = 1 and depth = 0 are indicated by green and red colors, respectively.


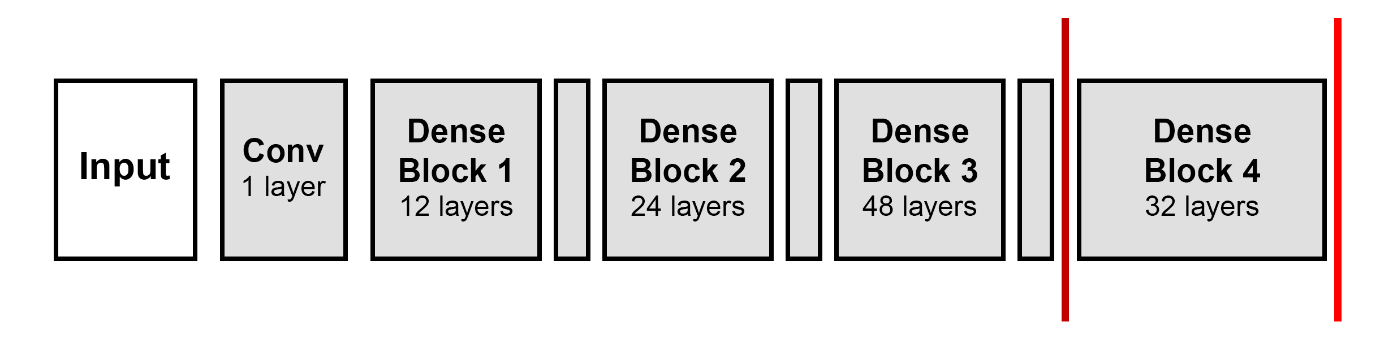


1. **NASNet Mobile**

The NASNet architecture is built upon two types of convolutional cells, whose structures have been optimally searched via a controller recursive neural network (RNN). “Normal Cells” are repeatable cells used to extract features, while “Reduction Cells” are designed for feature map reduction. The architecture is comprised of a single 3 × 3 convolutional layer for early feature extraction, followed by a series of normal cells and reduction cells as depicted in Figure 8. NASNet Mobile considered in our study refers to the “NASNet-A, 4 @ 1056” variant as denoted in the original paper, where four normal cells are repeated within a single group. For fine-tuning NASNet Mobile, we tuned only the last normal cell instance.


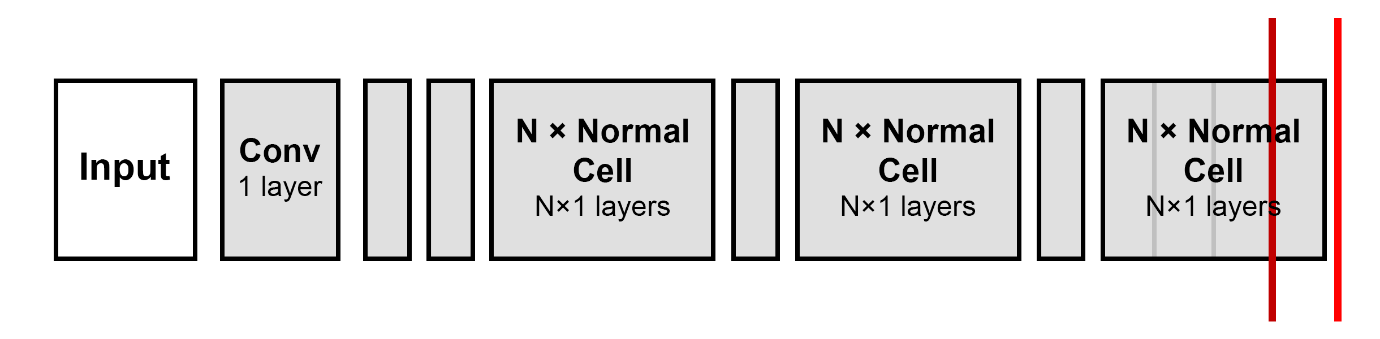


**Figure A8.** A simple illustration of the NASNet architecture, before global average pooling. The NASNet Mobile variant considered in our study (NASNet-A 4 @ 1056) has N = 4 normal repetitions within each group. The unlabeled blocks represent reduction cells. The lines indicate weight freezing boundaries, where depth = 1 and depth = 0 are indicated by green and red colors, respectively.

1. **Xception**

The Xception architecture uses separable convolutions and residual connections, similar to MobileNetV1 and ResNet. The architecture is comprised of an entry flow for early feature extraction, a middle flow repeated eight times, and an exit flow. All flows incorporate blocks of separable convolutions with residual connections. The middle flow consists of a single block of three separable convolutions (total of 6 convolutional layers) with a residual connection. For fine-tuning, we tuned all 8 layers in the exit flow.

**Figure A9.** A simple illustration of the Xception architecture, before global average pooling. The lines indicate weight freezing boundaries, where depth = 1 and depth = 0 are indicated by green and red colors, respectively.


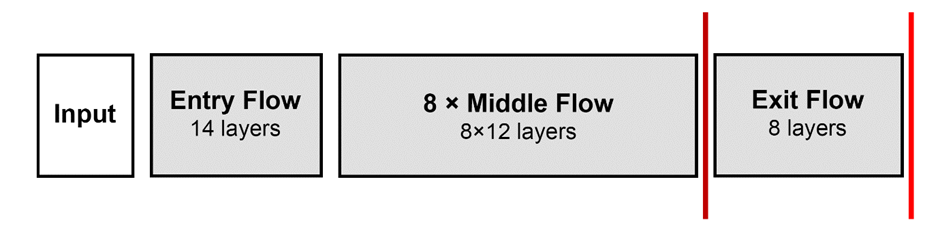


**REFERENCES**

1 Howard, A. G. *et al.* Mobilenets: Efficient convolutional neural networks for mobile vision applications. *arXiv preprint arXiv:1704.04861* (2017).

2 Sandler, M., Howard, A., Zhu, M., Zhmoginov, A. & Chen, L.-C. Mobilenetv2: Inverted residuals and linear bottlenecks. *Proceedings of the IEEE conference on computer vision and pattern recognition*, 4510-4520 (2018).

3 Simonyan, K. & Zisserman, A. Very deep convolutional networks for large-scale image recognition. *arXiv preprint arXiv:1409.1556* (2014).

4 Szegedy, C., Vanhoucke, V., Ioffe, S., Shlens, J. & Wojna, Z. Rethinking the inception architecture for computer vision. *Proceedings of the IEEE conference on computer vision and pattern recognition*, 2818-2826 (2016).

5 He, K., Zhang, X., Ren, S. & Sun, J. Identity mappings in deep residual networks. *European conference on computer vision*, 630-645, doi:doi.org/10.1007/978-3-319-46493-0_38 (2016).

6 Szegedy, C., Ioffe, S., Vanhoucke, V. & Alemi, A. A. Inception-v4, inception-ResNet and the impact of residual connections on learning. *Proceedings of the Thirty-First AAAI Conference on Artificial Intelligence*, 4278-4284 (2017).

7 Huang, G., Liu, Z., Van Der Maaten, L. & Weinberger, K. Q. Densely connected convolutional networks. *Proceedings of the IEEE conference on computer vision and pattern recognition*, 4700-4708 (2017).

8 Zoph, B., Vasudevan, V., Shlens, J. & Le, Q. V. Learning transferable architectures for scalable image recognition. *Proceedings of the IEEE conference on computer vision and pattern recognition*, 8697-8710 (2018).

9 Chollet, F. Xception: Deep learning with depthwise separable convolutions. *Proceedings of the IEEE conference on computer vision and pattern recognition*, 1251-1258 (2017).

10 Selvaraju, R. R. *et al.* Grad-cam: Visual explanations from deep networks via gradient-based localization. *Proceedings of the IEEE international conference on computer vision*, 618-626 (2017).
